# Case Report: The Sexual Experiences of Forensic Mental Health Patients

**DOI:** 10.3389/fpsyt.2021.651834

**Published:** 2021-04-09

**Authors:** Elnike Brand, Angela Ratsch, Edward Heffernan

**Affiliations:** ^1^Faculty of Medicine, University of Queensland, Brisbane, QLD, Australia; ^2^Wide Bay Hospital and Health Service, Research Services, Hervey Bay Hospital, Hervey Bay, QLD, Australia

**Keywords:** sexuality, sexual health, forensic mental health, forensic psychiatry, sexual rehabilitation

## Abstract

The recovery-based approach to forensic mental health rehabilitation is to support the patient to achieve a fulfilling life—a principle which should include achieving a fulfilling sexual life. This paper presents four vignettes from forensic mental health patients. The four cases demonstrate the omission, avoidance and then judgement by forensic mental health clinicians around the intimate and sensitive, yet important domain of the patient's sexual life. The cases illustrate that gap in the clinical domain and demonstrate the requirement for forensic mental health clinicians to have a greater awareness, acknowledgment, and assessment of their patient's sexuality and sexual health needs. Incorporating sexual health into standard clinical assessments will contribute to improved patient management in addition to supporting the principles of holistic forensic mental health recovery and rehabilitation.

## Introduction

An individual's sexuality is an inherent component of their everyday life experiences. The World Health Organization defines sexuality as, “…a central aspect of being human throughout life [and] encompasses sex, gender identities and roles, sexual orientation, eroticism, pleasure, intimacy, and reproduction. Sexuality is experienced and expressed in thoughts, fantasies, desires, beliefs, attitudes, values, behaviors, practices, roles, and relationships” (1). Yet despite this platform of normality and everydayness, juxtapositioned with the recovery principle of forensic mental health services focused on enabling everyday life activities (2), this individually important ordinary life construct is often not discussed as an element of standard forensic mental health clinical assessment. This assessment gap is a consequence of barriers by both the patient and the clinician around the subject, creating a sense of discomfort and a reluctance to discuss sexuality generally or concerns specifically (2–4).

The following cases share a common theme around the lack of appropriate identification, management and encouragement of appropriate and safe sexual experiences in a forensic mental health setting. Written informed consent was obtained from each of the patients. These patients were treated, involuntary, under the requirements of a Forensic Order Mental Health Act (Queensland) (5). None however had a history of sexual offending.

### Case 1

An elderly man with an established diagnosis of paranoid schizophrenia has spent the past 42 years in a high security forensic mental health facility. Despite his advanced age, he was alert and cognitively intact. He had an exceptional general knowledge but was guarded when questioned about his life and personal circumstances. He had no family support.

One evening, he spontaneously started to discuss his regrets in life with his treating psychiatrist. Surprisingly, it was not a life spent institutionalized, or the lack of opportunities for education or recreation, or even the loss of his family and social support network that this man regretted. This frail old man's only regret was not being able to have sex for the past 42 years.

He disclosed that nobody had ever questioned him about his sexual health needs during his 42 year inpatient stay, albeit he was questioned daily on every other single aspect of his life, his thoughts and his life experiences.

### Case 2

A middle-aged man returned from his first overnight leave from a forensic high security inpatient facility. He reported that he had “a very good night.” When prompted to elaborate, he shyly admitted that he had met a sex worker and engaged in a consensual and legal transaction. He revealed he had made this arrangement prior to his leave being approved. He fantasized, thought about, and planned this event for more than 2 years, without mentioning anything to any of his multidisciplinary treating team members. He stated that he was embarrassed and fearful that the team would not approve of this activity.

### Case 3

A fit, young, and otherwise healthy man with paranoid schizophrenia, in remission, engaged in auto-erotic asphyxiation on a weekly basis since puberty. He performed this activity in the privacy of his room when he knew he would not be disturbed, he was not distressed by this behavior, and did not pose any significant risk to himself or others. He therefore did not meet criteria for a paraphilic disorder. He declined any psychotherapy and wished to continue with this behavior. However, this became a major controversial point for the staff during his 3 year inpatient admission to the secure mental health facility. The “problem” remained following discharge to supervised accommodation.

### Case 4

A 34 year-old man treated for schizo-affective disorder under the requirements of a forensic order in the community remained non-compliant with his anti-psychotic medication. Different anti-psychotic treatments with different side effect profiles and methods of delivery were trialed without success. Following the establishment of therapeutic rapport and building a trusting professional alliance with his new psychiatrist, he reported that he experienced significant sexual side effects with medications, including retrograde ejaculation. He believed that he would lose the ability to have an erection and ejaculation and would not be able to have children, thus his non-compliance with his prescribed medications. He never had the courage to talk about these fears with the clinical team until prompted by the new psychiatrist.

## Discussion

As clinicians working in the field of sexology and forensic mental health, it became apparent to us that there was a gap in the identification and assessment of the sexuality and sexual health needs of forensic mental health patients Although all four case studies are male, the literature indicates that the sexual health needs of all mental health patients is impacted regardless of gender or sexual identity (6, 7). McMillan et al. (8) point out that on admission and during treatment, a patient's imminent mental health requirements and risks became the focus of their clinical care, and the concept of their sexual health, as part of their holistic care is diminished, i.e., their functioning as a whole sexual being is obviated.

### The Literature

Important information obtained from national surveys around sexuality and sexual health such as the Australian Study of Health and Relationships (9) and the British National Surveys of Sexual Attitudes and Lifestyles (10) aid policy makers in the development strategies for the improvement of sexual health in the general population (11). Unfortunately, these surveys often exclude institutionalized populations and underrepresent marginalized groups (12). Very few studies have evaluated the sexuality and sexual health of mental health patients despite the clinical knowledge that holistic sexual functioning is impacted by the combination of (a) the symptoms of mental illness, (b) social and interpersonal impairments, (c) institutionalization and (d) psychotropic medication (13). All of these factors are much more profound in forensic mental health patients. There are no large-scale studies that have gathered quantitative or qualitative data on the sexuality and sexual health needs of forensic mental health patients. This dearth in research then creates a vacuum in the information available for patients and clinicians to support evidence-based treatment decisions.

### The Clinical Discomfort Zone

Clinically it is important to explore mental health in relation to sexuality and sexual health for several reasons. Firstly, McCann et al. (14) systematic review highlighted that sexuality and sexual health cannot be achieved and maintained without respect for, and protection of, certain human rights. The fulfillment of sexuality and sexual health is tied to the extent to which human rights are respected, protected, and fulfilled and sexual rights are already recognized in international and regional human rights documents (1). Secondly, clinicians are generally unaware of the potential impact on sexual function of many mental health conditions (15). There is a bi-directional impact of mental health on sexual health; one's mental health affects one's sexual health, and one's sexual health affects one's mental health (16). This is particularly important given the young ages at which mental health problems tend to emerge and develop.

The third and fourth reasons are intertwined. Major mental illnesses are chronic and lifelong and affect all aspects of life, including sexual functioning. Sexual dysfunction impacts on quality of life and medication adherence. It is common knowledge that many medications used in the treatment of mental illness have a direct association with sexual dysfunction, decreased libido, erectile dysfunction, anorgasmia and decreased ejaculatory volume (17), yet these side effects are often overlooked, disregarded or forgotten in the decision making around medication choice. At the same time, this gap in clinical recognition and knowledge is compounded by patients' reluctance to self-report sexual dysfunction symptoms despite sexual dysfunction having a considerable impact on quality of life and adherence to antipsychotic medications (18–20). Evans et al. (4) points out that the barriers of addressing sexual health needs from a patient's perspective include clinical setting, communication difficulties, the possible consequence of addressing this with clinicians and medication related side effects. This is more profound in forensic patients, who often reside involuntarily for prolonged periods in a confined and strictly regulated hospital environment, where the clinical setting does not support or promote sexual experiences (21).

McCann et al. (14) recognized that the assessment of sexuality is often neglected by mental health clinicians while acknowledging the challenges of institutionalization, risk, stigma, communication, and clinical setting. Urry et al. (2) concluded that mental health clinicians often omit the assessment of their patient's sexual health as it is a “peripheral issue” and “not pragmatic to bring up sexual health.”

## Conclusion

While it is widely acknowledged that understanding sexuality and sexual health needs are important in the overall recovery of mental health patients, there is a reluctance by clinicians and patients alike to explore the patient's sexual health needs. This is even more evident in the field of forensic mental health where treatment and management often focus on imminent mental health care needs and risks and spans many years. Incorporating sexuality and sexual health into standard clinical assessments will contribute to supporting holistic forensic mental health recovery and improve quality of life.

## Recommendations

There are data around normal sexual experiences, and some emerging data on the sexual experiences of mental health patients and the barriers they encounter, both from a patient and a clinician perspective. Given the dearth of quantitative and comparison information in forensic mental health patients, the authors recommend the development of research at the intersection of forensic mental health and sexology around “*What is the sexual development, sexual experiences, sexual knowledge and sexual health of forensic mental health patients?”*

The benefits of such research are 2-fold. The first will be the development of an understanding of the sexuality and sexual health of forensic mental health patients. The second will inform the development of evidence-based intervention programs to enable this vulnerable cohort to engage in socially appropriate experiences to achieve a fulfilling sexual life. Given there is an absence of information in relation to this cohort and this theme, it is recommended that the research program should use mixed methods to assess the sexual development, experiences, knowledge and sexual health of forensic mental health patients.

The conduct of research around this theme will align with the recovery principles in forensic mental health. From the perspective of the individual with mental illness, recovery means gaining and retaining hope, understanding of one's abilities and disabilities, engagement in an active life, personal autonomy, social identity, meaning and purpose in life, and a positive sense of self. While patients with mental health illnesses often receive individual or group treatment for a range of other health needs which are discrete from their mental health treatment, for example drug and alcohol, there is an absence of support structures for people whose mental illness has affected their sexuality. In addition, there is also an absence of any individualized approach that clinicians can use to support these patients to achieve a satisfactory outcome. Research in this theme area will contribute to knowledge and evidence to support and foster recovery.

## Data Availability Statement

The original contributions presented in the study are included in the article/supplementary material, further inquiries can be directed to the corresponding author/s.

## Ethics Statement

The studies involving human participants were reviewed and approved by Royal Brisbane and Women's hospital human research ethics committee. The patients/participants provided their written informed consent to participate in this study.

## Author Contributions

EB conceived the case study and wrote the initial manuscript. AR and EH edited the manuscript. All authors contributed to the article and approved the submitted version.

## Conflict of Interest

The authors declare that the research was conducted in the absence of any commercial or financial relationships that could be construed as a potential conflict of interest.
